# 3D-printed bolus ensures the precise postmastectomy chest wall radiation therapy for breast cancer

**DOI:** 10.3389/fonc.2022.964455

**Published:** 2022-09-02

**Authors:** Xiran Wang, Jianling Zhao, Zhongzheng Xiang, Xuetao Wang, Yuanyuan Zeng, Ting Luo, Xi Yan, Zhuang Zhang, Feng Wang, Lei Liu

**Affiliations:** ^1^ Department of Head and Neck and Mammary Oncology, West China Hospital, Sichuan University, Chengdu, China; ^2^ Department of Radiotherapy, West China Hospital, Sichuan University, Chengdu, China; ^3^ Clinical Research Center for Breast, West China Hospital, Sichuan University, Chengdu, China; ^4^ State Key Laboratory of Oral Diseases, West China Hospital of Stomatology, Sichuan University, Chengdu, China

**Keywords:** 3D-printed bolus, breast cancer, PMRT, dosimetry, radiation dermatitis

## Abstract

**Purpose:**

To investigate the values of a 3D-printed bolus ensuring the precise postmastectomy chest wall radiation therapy for breast cancer.

**Methods and materials:**

In the preclinical study on the anthropomorphic phantom, the 3D-printed bolus was used for dosimetry and fitness evaluation. The dosimetric parameters of planning target volume (PTV) were assessed, including D_min_, D_max_, D_mean_, D_95%_, homogeneity index (HI), conformity index (CI), and organs at risk (OARs). The absolute percentage differences (|%diff|) between the theory and fact skin dose were also estimated, and the follow-up was conducted for potential skin side effects.

**Results:**

In preclinical studies, a 3D-printed bolus can better ensure the radiation coverage of PTV (HI 0.05, CI 99.91%), the dose accuracy (|%diff| 0.99%), and skin fitness (mean air gap 1.01 mm). Of the 27 eligible patients, we evaluated the radiation dose parameter (median(min–max): D_min_ 4967(4789–5099) cGy, D_max_ 5447(5369–5589) cGy, D_mean_ 5236(5171–5323) cGy, D_95%_ 5053(4936–5156) cGy, HI 0.07 (0.06–0.17), and CI 99.94% (97.41%–100%)) and assessed the dose of OARs (ipsilateral lung: D_mean_ 1341(1208–1385) cGy, V_5_ 48.06%(39.75%–48.97%), V_20_ 24.55%(21.58%–26.93%), V_30_ 18.40%(15.96%–19.16%); heart: D_mean_ 339(138–640) cGy, V_30_ 1.10%(0%–6.14%), V_40_ 0.38%(0%–4.39%); spinal cord PRV: D_max_ 639(389–898) cGy). The skin doses *in vivo* were D_theory_ 208.85(203.16–212.53) cGy, D_fact_ 209.53(204.14–214.42) cGy, and |%diff| 1.77% (0.89–2.94%). Of the 360 patients enrolled in the skin side effect follow-up study (including the above 27 patients), grade 1 was the most common toxicity (321, 89.2%), some of which progressing to grade 2 or grade 3 (32, 8.9% or 7, 1.9%); the radiotherapy interruption rate was 1.1%.

**Conclusion:**

A 3D-printed bolus can guarantee the precise radiation dose on skin surface, good fitness to skin, and controllable acute skin toxicity, which possesses a great clinical application value in postmastectomy chest call radiation therapy for breast cancer.

## Introduction

Breast cancer is the most common carcinoma that accounts for 30% of female cancers according to the latest statistics conducted by the International Agency for Research on Cancer, with approximately 2.3 million new cases in 2020 (1, 2). Comprehensive treatments including surgery, chemotherapy, radiotherapy, endocrine therapy, and biotherapy are the main therapeutic modalities for breast cancer. Previous studies have shown the mastectomy rates remaining between 30% and 40% (3). Post-mastectomy radiotherapy (PMRT) is associated with a better local control and overall survival benefit in patients with unfavorable pathologic features (4–8).

During the process of radiotherapy, the maximum radiation dose of high-energy X-ray beams can be reached only after they enter the human tissue with a certain depth, which is named built-up effect or skin sparing effect (9–12). Thus, a tissue-equivalent bolus needs to be placed on the skin’s surface, aiming to reduce the risk of local recurrence and improve the long-term survival rate in PMRT, such as wet gauze, paraffin wax, thermoplastic board, and so on (13). Although the use of a bolus was controversial due to skin toxicity, a worldwide e-mail survey showed that 82% of Americans and 65% of Australasians were likely to always use a bolus when delivering PMRT. Europeans were significantly more likely to use a bolus for specific indications (p < 0.0001) (14–21). Meanwhile, the bolus thickness and frequency of use also vary considerably between centers and are closely related to the incidence and severity of radiation dermatitis. Vu et al. found that 35% of respondents used a <10 mm bolus, most of which (89%) used a thickness of 5 mm (with responses varying from 3 to 8 mm), and the occurrence rate of severe skin reactions was 5%–30% (22). Spierer et al. found that 63.6% developed grade 3–4 skin toxicity in a follow-up study of 118 patients with the daily use of a bolus (the radiotherapy interruption rate was 28%) (23). Pignol et al. recorded acute skin toxicities of 257 patients who received PMRT; the rate of grade 3 toxicity was as high as 47% for the daily use of a 5–10 mm bolus versus only 26% for once every other day use (p < 0.001) (16). Another study showed that there was no observed adverse effect by adding a 5 mm bolus on alternate days in the median follow-up of 3.7 years (range 1–6.6 years) (24). In addition to the effect of cumulative dose on the skin surface, smoking history (p = 0.03), radiation energy (p = 0.04), human race (p = 0.031), BMI (p = 0.043), and postmenopausal status (p = 0.004) were all correlated (14, 16, 25). Thus, the National Comprehensive Cancer Network Guidelines (NCCN, version 4.2022) and the European Society for Radiotherapy & Oncology (ESTRO) recommend that special consideration should be given to the daily use of a 3–5 mm bolus in the setting of PMRT to select cases, especially for inflammatory breast cancer, skin involvement (T_4b-d_), and positive anterior margin (20, 24, 25).

However, due to the irregular chest wall shape and surgical scar, it is difficult to make the commercial bolus conform perfectively with the skin; in addition, it is also easy to be deformed during radiotherapy, which usually causes air gaps between the bolus and skin (26–29). Some studies have shown that these gaps can lead to inadequate or inhomogeneous radiation doses to the skin, which may further reduce the effect of PMRT (30–34). The emerging three-dimensional (3D) printing technology offers alternative fabrication ways for an ideal patient-specific bolus, which can further optimize the effectiveness of radiotherapy (35–40). Previous studies have revealed that the patient-specific bolus reduces unnecessary irradiation to the healthy normal tissues and improves the conformity of radiation distribution for patients with irregular surface contours and varying target depths (35, 41–46). Even though a 3D-printed bolus has been gradually applied in superficial tumor radiotherapy, the clinical application of PMRT still remains spare (26, 28). This study used the patient-specific 3D-printed bolus for PMRT and evaluated the dosimetric characteristics, skin fitness, and skin adverse effects of the 3D-printed bolus, hoping to achieve improved results by ensuring a more precise radiotherapy for breast cancer patients.

## Material and methods

### 3D-printed bolus design and fabrication

**Figure 1:**
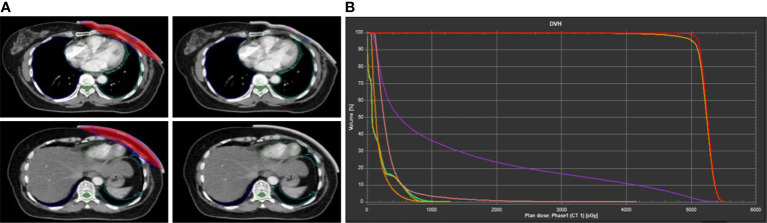
Example of dose distribution of 3D-printed bolus in treatment planning system (TPS). **(A)** Delineation of radiotherapy target area. **(B)** The dose–volume histogram (DVH) curve.

The desired bolus area for radiotherapy was marked on the anthropomorphic phantom or patient. The chest contour was created based on the computed tomography (CT) scan, which is then expanded by the desired thickness of the bolus and subtracted from the expansion. CT images in the general digital imaging and communications in medicine (DICOM) format were exported as a stereolithography (STL) file, which was loaded into a 3D-modeling software (Mimics 10.01) to create a patient-specific bolus ( Supplementary Table 1 and **Figure 1**). It should be noted that in order to reduce the positioning error, we developed the positioning fixator connecting the vacuum bag and the bolus; the manufacturing process of the 3D-printed bolus (thickness: 5 mm) is shown in Supplementary Figure 2.

In order to ensure that the 3D-printed bolus highly fits with the skin to further assure the radiotherapy quality, the following aspects were noted: first, a connecting fixing device was designed between the axillary side of the bolus and the vacuum bag to prevent the bolus from shifting; second, cone beam computed tomography (CBCT) was daily used to verify the reproducibility of bolus placement; third, the 3D-printed bolus was remade if necessary.

### Participant population

We selected patients according to the following criteria: women aged 18–70 years who underwent radical mastectomy and primary chest wall radiotherapy, patients with _P_T_3-4_ or _P_N_2-3_ stage, one to three axillary lymph nodes positive at the _P_T_1-2_ stage with high-risk factors (age ≤40 years, estrogen receptor and progesterone receptor negative (ER-/PR-), human epidermal growth factor receptor 2 overexpression (HER2+++), histologic grade III (G3), lymphovascular invasion (LVI), etc.). Patients receiving radiotherapy with a commercially available bolus or without a bolus, with recurrent or metastatic disease, or previously treated, were excluded.

### Dosimetric evaluation

A radiotherapist delineated the clinical target volume (CTV): the upper border was the clinically visible/palpable one and not exceeding the sternoclavicular joint (~2nd rib), the lower border was the inferior margin of the contralateral breast on CT, the anterior border extended to the skin, the posterior border included the pectoralis muscles and ribs, and the medial and lateral borders were sternum and mid-axillary line (excluding latissimus dorsi) and the organs at risk (OARs) in the RayStation treatment planning system (TPS) (version4.7.5; RaySearch Laboratories AB, Stockholm, Sweden) (47, 48). The intensity modulated radiation therapy (IMRT) within six-field irradiations was used in PMRT. The inner and outer tangential field was used in the chest wall, and the angle of increasing field was within ±15° based on the spatial relationship between the target area and the organs at risk. Pairs of penetrating field were added in the locking segment based on the radiating field of the chest wall. The angles of the radiating field of the left and right breasts were 340° and 160°, respectively, and 20° and 200°, respectively. Number of segments: 48. The maximum field area is 4 mm^2^, and the maximum field hop number is 4 MU. All treatment plans are designed in the RayStation TPS (**Figure 1** **)**, with a 6 MV photon beam and a collapsed cone algorithm. The dose grid size is 0.3 * 0.3 * 0.3 cm. The prescribed doses were PCTVsc (the supra- and infra-clavicular regions) and PCTVcw (the ipsilateral chest wall) 50 Gy/25 f, and doses were normalized to at least 95% target volume meeting the prescribed dose requirements (49–51). The dosimetric parameters of the planning target volume (PTV: defined as the CTVs with a 5 mm margin) were evaluated as follows: D_min_, D_max_, D_mean_, D_95%_, homogeneity index (HI = (D_2%_-D_98%_)/D_50%_), conformity index (CI), absolute percentage differences (|%diff|=|100x (D_fact_- D_theory_)/D_theory_|) for single fraction; OARs: ipsilateral lung (D_mean_, V_5_, V_20_, V_30_), heart (D_mean_, V_30_, V_40_), and spinal cord PRV(D_max_) (31, 49, 52).

### *In vivo* skin dose measurement

GafChromic EBT3 (International Specialty Products, Wayne, NJ, USA) had been proven to be suitable for absorbed dose measurement in radiotherapy (53), which was used in our study due to its thin structures, easily cutting to small size and near-tissue equivalence. To accurately position the EBT3 films, beam’s eye view (BEV) at a gantry angle of 0 degree with PTV and body contours on show was printed on a paper with a scale of 1:1 to a real patient. The PTV contour was divided into eight sub-regions by four rows and two columns, with rows toward left–right and columns toward cranial–caudal directions. Eight 3 × 2 cm^2^ rectangles were drawn and marked with numbers 1, 2,…, 8 in the center of each sub-region, respectively (Supplementary Figure 3). EBT3 film pieces with a fixed size of 3 × 2 cm^2^ were cut from the same batch. For each patient, eight film pieces coded with numbers 1, 2,…, 8 were taped on the chest wall at the positions corresponding to the eight rectangles and covered by the 3D-printed bolus. For the sub-region where the patient’s surface was very unsmooth, particularly in the region near the axilla, the 3 × 2 cm^2^ film piece was replaced by a smaller one with a size of 2 × 1.5 cm^2^.

Every patient’s irradiated films with two reference films together were scanned by an Epson 11000XL scanner 24 h after irradiation. The two reference films—one was unexposed and the other was exposed to a known dose immediately after *in vivo* measurement—were used to rescale the calibration function to fit the responses of that specific scan. Software FilmQA Pro 2016 was used to analyze the measurement results. The film absorbed dose was achieved by averaging the reading of a region of interest (ROI) with 1 × 1 cm^2^ at the center of each film piece.

**Figure 2:**
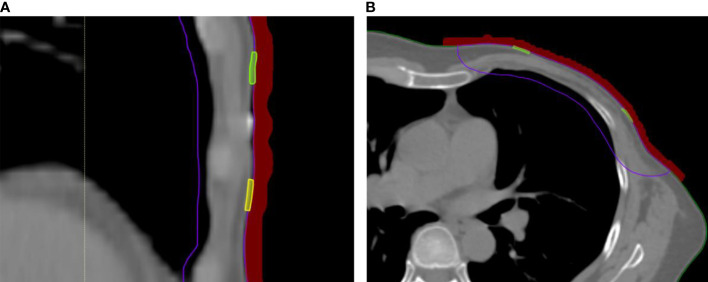
*In-vivo* skin doses measurement in RayStation TPS. **(A)** Coronal scan with bolus covering small film. **(B)** Cross-sectional scan with bolus covering small film.

The calculated surface doses were obtained in TPS. For every patient, eight ROIs in the center of each sub-region with a size of 1 × 0.1 cm between the 3D-printed bolus and the patient skin across three-slice CT images were contoured (an example of the ROI contour is shown in **Figure 2**). The average dose of each ROI was recorded and compared with the measurement dose.

### Skin toxicity

All patients referred for PMRT were visited weekly during and after 2–4 weeks of radiotherapy to assess and record skin toxicities. To ensure consistency and accuracy in the classification of acute skin side effect, the follow-up photographs of the skin (Supplementary Figure 4) were evaluated by two or three radiotherapists to determine the grading (according to the Radiation Therapy Oncology Group (RTOG)) (54). For cases with uncertain grading results, a dermatological consultation with the patient might be requested. The occurring time of skin side effect including dry or moist desquamation and the degree of erythema were evaluated. Based on the RTOG classification, the main difference between grades 2 and 3 was the presence of moist desquamation and tenderness, while grade 4 was defined as necrosis, ulceration, or bleeding.

During radiotherapy, skin care includes the following: keeping the irradiated chest wall dry, avoiding skin scratching, medical ray protection sprays, and corticosteroids or topical dressings used for excessive inflammation; antibiotics were used when necessary.

## Result

### Preclinical evaluation

The theoretical radiation dose of the chest wall reached the targeted values (mean value): D_min_ 4932 cGy, D_max_ 5259 cGy, D_mean_ 5131 cGy, D_95%_ 5021 cGy, HI 0.05, CI 99.91%. Meanwhile, there was a strict limit on the OARs in TPS: the D_mean_ values of the ipsilateral lung and heart were 1017 and 438 cGy, respectively, the D_max_ value of the spinal cord PRV was 88 cGy (**Table 1**). The mean D_fact_ and D_theory_ of the skin surface were 204.59 and 204.73 cGy, respectively, and the mean |%diff| was 0.99%. In addition, we also observed that the 3D-printed bolus was highly attached to the skin, and the mean air gap at the dosimetry point on the skin surface was only 1.01 mm (Supplementary Table 2).

**Table 1:** Dosimetry evaluation of anthropomorphic phantom with 3D-printed bolus .

PTV	Mean value	OARs		Mean value
*D_min_, cGy	4932	Ipsilateral lung	D_mean_, cGy	1017
D_max_, cGy	5259		V_5_	38.80%
D_mean,_ cGy	5131		V_20_	19.56%
D_95%_, cGy	5021		V_30_	12.54%
D_2%_, cGy	5251	Heart	D_mean_, cGy	438
D_98%_, cGy	4977		V_30_	1.42%
D_50%_, cGy	5134		V40	0.30%
HI	0.05	Spinal Cord	D_max_, cGy	81
CI	99.91%	Spinal Cord PRV	D_max_, cGy	88

3D, three-dimensional; PTV, planning target volume; HI, homogeneity index ((D_2%_-D_98%_)/D_50%_); CI, conformity index; PRV, planning organs at risk volume.

*D_min_, minimum dose of the target volume; D_max_, maximum dose of the target volume; D_mean_, mean dose of the target volume; D_95%_, the dose that covers 95% of the target volume.

### Clinical evaluation

#### Patient population

Finally, we totally involved 360 patients in this study from October 2019 to July 2021 (**Table 2**), in which 27 patients were selected to study dosimetric parameters; the median age was 49 (24–70) years old. The lesions were mostly in the left breast (199 of 360, 55.3%). There were 24.7% (89 of 360) or 50.3% (181 of 360) of the patients with advanced pathologic stages T_3-4_ or N_2-3_. In addition, 39.4% (142 of 360) of the patients with early pT_1-2_N_1_ had at least one (85 of 142, 59.9%) and up to four (1 of 142, 0.7%) risk factors, including age ≤40 years (20 patients), ER-/PR- (42 patients), HER2+++ (59 patients), G3 (70 patients), and LVI (22 patients), in which 42 patients had two risk factors and 14 patients had three risk factors. In the tumor-node-metastasis (TNM)-based staging (8th Edition of the American Joint Committee on Cancer (AJCC) publications), patients with stage III disease accounted for the majority (217 of 360, 60.3%), in which 27.5% (99 of 360), 8.9% (32 of 360), and 23.9% (86 of 360) of the patients had stage A, B, and C, respectively. The next largest number of patients belonging to IIB was 29.6% (107 of 360). Postoperative breast reconstruction was rare, only 11.4% (41 of 360) of the patients; the rest of the patients did not undergo breast reconstruction (319 of 360, 88.6%).

**Table 2:** Patient characteristics.

	Skin follow-up (n = 360)
Age (years)	
Median (range)	49 (24–70)
Lesion sites	
Left, n (%)	199 (55.3%)
Right, n (%)	161 (44.7%)
pT_3-4_-stage	
T_3_, n (%)	41 (11.4%)
T_4_, n (%)	48 (13.3%)
pN_2-3_-stage	
N_2_, n (%)	95 (26.4%)
N_3_, n (%)	86 (23.9%)
pT_1-2_N_1_, n (%)	142 (39.4%)
age ≤40 y, n	20
ER-/PR-, n	42
HER2+++, n	59
G3, n	70
LVI, n	22
Tumor stage	
IIA, n (%)	36 (10.0%)
IIB, n (%)	107 (29.7%)
IIIA, n (%)	99 (27.5%)
IIIB, n (%)	32 (8.9%)
IIIC, n (%)	86 (23.9%)
Breast Reconstruction	
With, n (%)	41 (11.4%)
Without, n (%)	319 (88.6%)

pT-stage, pathologic tumor stages; pN-stage, pathologic node stages; G3, histologic grade III; LVI, lymphovascular invasion; ER-,estrogen receptor negative; PR-, progesterone receptor negative; HER2+++, human epidermal growth factor receptor 2 overexpression.

### Dosimetric parameters evaluation in 27 patients

The dose coverage in the target area met the prescription dose requirements in TPS: median dose(range): D_min_ 4967 (4789–5099) cGy, D_max_ 5447(5369–5589) cGy, and D_mean_ 5236(5171–5323) cGy; D_95%_ of the target volume ranged from 4936 to 5156 cGy. The CI and HI were 99.94% (97.41%–100%) and 0.07(0.06–0.17), respectively (Supplementary Table 3). The actual radiation dose on the skin surface was very close to the theory value; the median theoretical and actual radiation doses were 208.85(203.16–212.53) cGy and 209.53(204.14–214.42) cGy, respectively, and the |%diff| ranged between 0.89% and 2.94%, with median 1.77% (**Table 3**). In addition, the dose of OARs is illustrated in Supplementary Table 4. The median D_mean_ of the ipsilateral lung was 1341(1208–1385) cGy; the V_5%_, V_20%_ and V_30%_ of the target volume were 48.06% (39.75%–48.97%), 24.55% (21.58%–26.93%), and 18.40% (15.96%–19.16%), respectively. The D_mean_ of the heart was 339 (138–640) cGy, with V_30_ 1.10% (0%–6.14%) and V_40_ 0.38% (0%–4.39%). The median D_max_ of the spinal cord PRV was 639(389–898) cGy.

**Table 3:** Dose accuracy verification on skin surface of the 27 patients.

	*D_fact_ (cGy)	D_theory_ (cGy)	|%diff| (%)
P1	213.30	211.31	0.94
P2	209.53	208.52	0.48
P3	206.37	203.16	1.58
P4	208.40	205.86	1.23
P5	211.01	209.38	0.78
P6	207.71	208.41	0.34
P7	209.57	210.86	0.61
P8	205.29	204.55	0.36
P9	210.66	212.53	0.88
P10	212.18	209.03	1.51
P11	211.63	209.92	0.81
P12	210.70	211.09	0.18
P13	208.51	207.75	0.37
P14	204.44	204.83	0.19
P15	204.99	206.63	0.79
P16	209.53	207.75	0.86
P17	208.46	208.80	0.16
P18	207.51	206.73	0.38
P19	214.11	211.01	1.47
P20	212.51	208.42	1.96
P21	209.61	208.92	0.33
P22	212.90	208.59	2.07
P23	204.14	206.03	0.92
P24	211.46	211.59	0.06
P25	205.04	209.14	1.96
P26	208.94	210.67	0.82
P27	214.42	211.36	1.45
Median (min-max)	209.53 (204.14-214.42)	208.85 (203.16-212.53)	1.77 (0.89-2.94)

*D_theory_, theoretical radiation dose for chest wall skin; D_fact_, fact radiation dose for chest wall skin; |%diff| (the absolute percentage difference=|100x (D_fact_- D_theory_)/D_theory_|), the absolute differences between theoretical and fact doses at the skin surface.

### Skin toxicity

All the 360 patients were followed up for skin toxicity study during the radiotherapy (**Table 4**). The most common skin toxicity was grade 1 (321 of 360, 89.2%), presenting as faint erythema (229 of 321,71.4%) or dry desquamation (54 of 321, 16.8%) or both (38 of 321, 11.8%). With the accumulation of radiation dose (especially during 21–25 fractions), the number of patients with the above symptoms was also gradually increasing (84 of 229, 36.7%; 24 of 54, 44.5%; 21 of 38, 55.3%). With a small number of patients progressing to grade 2 (32 of 360, 8.9%), all patients presented moderate erythema (MER), in which 56.2% or 28.2% of the patients had accompanied patchy moist desquamation (PMD) (18 of 32) or moderate edema (MED) (9 of 32) that occurred after 21 fractions (14 of 18, 77.8%; 7 of 9, 77.8%); others presented large areas of MER and MED, with PMD at the folds of the skin, but the number of patients was relatively small (5 of 32, 15.6%). The incidence of grade 3 was relatively low (7 of 360, 1.9%); most patients present confluent moist desquamation (CMD) and pitting edema (PE) (5 of 7, 71.4%); treatment was discontinued in four patients because they developed during radiotherapy (1.1%). There was no grade 4 occurrence (0 of 360). The most severe reactions usually occur in the 2–4 weeks after completion of radiotherapy treatment (**Table 5**). The incidence of grades 2–4 acute radiation dermatitis was 41.67%, of which 64.7% were grade 2 that presented as complex lesions (moderate erythema with edema, patchy moist desquamation at the skin fold). Grade 4 events (mainly ulcers) occurred in 4.4% of the patients, and all got well again after topical corticosteroids and dressing therapy combined with antibiotics.

**Table 4:** Skin toxicity during radiotherapy.

	≤10f	11-15f	16-20f	21-25f	Total patient
Grade 1, n (%)					321(89.2%)
Faint erythema, n (%)	32 (13.9%)	48 (20.9%)	65 (28.5%)	84 (36.7%)	229 (71.4%)
Dry desquamation, n (%)	6 (11.1%)	7 (12.9%)	17 (31.5%)	24 (44.5%)	54 (16.8%)
Both, n (%)	1 (2.6%)	4 (10.5%)	12 (31.6%)	21 (55.3%)	38 (11.8%)
Grade 2, n (%)					32(8.9%)
PMD+MER, n (%)	0	0	4 (22.2%)	14 (77.8%)	18 (56.2%)
MER+MED, n (%)	0	0	2 (22.2%)	7 (77.8%)	9 (28.2%)
PMD+MER+MED, n (%)	0	0	0	5 (100%)	5 (15.6%)
Grade 3, n (%)					7 (1.9%)
PE, n (%)	0	0	1 (50%)	1 (50%)	2 (28.6%)
PE+CMD, n (%)	0	0	0	5 (100%)	5 (71.4%)
Grade 4, n (%)	0	0	0	0	0
Treatment interruption, n (%)	0	0	2 (50%)	2 (50%)	4 (1.1%)

PMD, patchy moist desquamation; MER, moderate erythema; MED, moderate edema; CMD, confluent moist desquamation; PE, pitting edema.

**Table 5:** Skin toxicity during and after 2–4 weeks of radiotherapy.

	During the radiotherapy	2–4 weeks after radiotherapy
Grade 1	321 (89.12%)	210 (58.33%)
Grade 2	32 (8.89%)	97 (26.94%)
Grade 3	7 (1.94%)	37 (10.28%)
Grade 4	0	16 (4.4%)

## Discussion

In this study, the largest of its kind, we illustrated that the use of the 3D-printed bolus brought many advantages in postmastectomy chest wall radiation therapy for breast cancer, such as reducing the air gaps between the bolus and the skin, improving the dose uniformity, and ensuring the skin surface radiation dose, which might further guarantee the precise PMRT for breast cancer.

Generally speaking, unwanted air gaps lead to an inadequate or inhomogeneous radiation dose, which causes a considerable difficulty for the precise postmastectomy chest wall radiation therapy for breast cancer (30). Butson and Khan et al. reported that the dose for high-energy X-ray beam was decreased by up to 4% and 10% because of 4 and 10 mm air gaps, respectively (33, 55). Zhao et al. reported that 11 mm air gap under the commercial bolus obviously decreased the skin surface dose by about 2% (56). Similarly, James L. Robar et al. found that the air gaps of more than 5 mm were decreased from 30% (commercial bolus) to 13% (3D-printed bolus) (p < 0.0003), and the maximum air gaps diminished from 5 ± 3 to 3 ± 3 mm (26). Our study showed that the unwanted air gaps were reduced to as low as 1.01 mm contacting better with the patient’s irregular skin surface. Accurate fitting of the bolus to the patient skin is important, and thus, our study pointed out that customized 3D-printed boluses with better fitting are suitable for clinical applications.

Furthermore, our personalized 3D-printed bolus provided an optimal dose distribution, with HI lower than 0.07 and CI >99.9%. However, the HI of the commercial bolus was 0.15 in the study of Zhang et al., which greatly reduced the effectiveness of radiotherapy (26). Hou and Park et al. also found that the 3D-printed bolus improved dose uniformity by 45% and improved the precision of the dose absorbed by the chest wall to 3% (28, 57). However, the HI and CI in their studies still did not reach a lower value. In our study, we used IMRT technology and a positioning fixation device to reduce positioning error, improve the dose uniformity, ensure skin surface radiation dose, and maximize precision radiotherapy. In addition, the actual radiotherapy dose of the skin was almost close to the theoretical dose (D_theory_ 208.85 (203.16-212.53) cGy, D_fact_ 209.53 (204.14-214.42) cGy, |%diff| 1.77% (0.89-2.94%)). This result was obviously better than the traditional bolus in the study of Park et al. whose |%diff| was 4.43% (28).

It is worthy to note that although the 3D-printed chest wall bolus has obvious dosimetric advantages, radiodermatitis is one of the distressing side effects that manifested as erythema or moist desquamation even. Although most radiodermatitis is reversible, it commonly causes discomfort and may bring about treatment interruption. Therefore, we have taken some measures to further reduce the incidence of radioactive dermatitis, such as skin care education for patients before radiotherapy, including keeping the irradiated chest wall dry; avoiding skin scratching; and using medical ray protection sprays, corticosteroids, or topical dressings appropriately; antibiotics were used when necessary, and so on. In this current study, the skin side effect incidences of grade 1 (321 of 360, 89.2%), grade 2 (32 of 360, 8.9%), and grade 3 (7 of 360, 1.9%) were controllable during the radiotherapy, which was similar to the incidence of radiation dermatitis caused by a traditional bolus reported by Anderson and Tieu et al. whose ≥2 grade dermatitis was 9%–24% (15, 58). However, in our study, fewer patients (4 of 360, 1.1%) had to discontinue treatment because of more unacceptable skin toxicity than Tieu’s (20 of 254, 7.9%) (15). We speculated that it was the patient skin care education before radiotherapy and strict follow-up that, to a certain extent, guaranteed the patient’s compliance to the whole treatment.

However, our study presents several limitations. Firstly, the study was a single-arm, single-center clinical study; the results need to be further verified in a multicenter study in the future. Secondly, since this study paid more attention to the 3D-printed bolus ensuring the precise postmastectomy chest wall radiation therapy for breast cancer, quality of patient life assessments may have been overlooked. Thirdly, the follow-up time was only limited in the radiation period, and there needs to be longer follow-up time for the 3D-printed bolus’ effect on locoregional control and patient survival.

## Conclusion

The new 3D-printed chest wall bolus owns a high degree of personalization, good radiation dosimetric advantages, and controllable skin toxicity, which has a relatively high clinical application value. In the future, long-term follow-up will be continued to evaluate the patient’s local recurrence and survival so as to comprehensively evaluate the efficacy of the 3D-printed bolus in PMRT. Meanwhile, we will explore new 3D-printed bolus materials with higher quality and lower price, and seek the best application times to ensure the curative effect of radiotherapy.

## Data availability statement

The original contributions presented in the study are included in the article/supplementary material. Further inquiries can be directed to the corresponding author.

## Ethics statement

Written informed consent was obtained from the individual(s) for the publication of any potentially identifiable images or data included in this article.

## Author contributions

XRW, JLZ and ZZX who write the manuscript and are responsible for statistical analysis have contributed equally to this work. XTW and YYZ are responsible for guiding the writing the paper. TL, XY, ZZ and FW take charge of recruiting patients. Liu Lei is responsible for the overall revision. All authors contribute to the article and approve the submitted version.

## Funding

This research was supported by Ministry of Science and Technology of Sichuan Province (Grant No. 2019YFS0362).

## Acknowledgments

We thank our colleagues for the critical reading of the manuscript.

## Conflict of interest

The authors declare that the research was conducted in the absence of any commercial or financial relationships that could be construed as a potential conflict of interest.

## Publisher’s note

All claims expressed in this article are solely those of the authors and do not necessarily represent those of their affiliated organizations, or those of the publisher, the editors and the reviewers. Any product that may be evaluated in this article, or claim that may be made by its manufacturer, is not guaranteed or endorsed by the publisher.
